# Advising and limiting medical treatment during phone consultation: a prospective multicentre study in HEMS settings

**DOI:** 10.1186/s13049-022-01002-8

**Published:** 2022-03-09

**Authors:** Heidi Kangasniemi, Piritta Setälä, Heini Huhtala, Anna Olkinuora, Antti Kämäräinen, Ilkka Virkkunen, Joonas Tirkkonen, Arvi Yli-Hankala, Esa Jämsen, Sanna Hoppu

**Affiliations:** 1 Research and Development Unit, FinnHEMS Ltd, WTC Helsinki Airport, Lentäjäntie 3, 01530 Vantaa, Finland; 2 Division of Anaesthesiology, Department of Perioperative, Intensive Care and Pain Medicine, HUS University of Helsinki and Helsinki University Hospital, Meilahti Tower Hospital, Haartmaninkatu 3, 00029 Helsinki, Finland; 3 Faculty of Medicine and Health Technology, Tampere University, 33014 Tampere, Finland; 4 Emergency Medical Services, Centre for Prehospital Emergency Care, Department of Emergency, Anaesthesia and Pain Medicine, Tampere University Hospital, P.O. Box 2000, 33521 Tampere, Finland; 5 Faculty of Social Sciences, Tampere University, P.O. Box 100, 33014 Tampere, Finland; 6 Department of Emergency Medicine, Hyvinkää Hospital, 05850 Hyvinkää, Finland; 7 Department of Intensive Care Medicine, Tampere University Hospital, P.O. Box 2000, 33521 Tampere, Finland; 8 Department of Emergency, Anaesthesia and Pain Medicine, Tampere University Hospital, P.O. Box 2000, 33521 Tampere, Finland; 9 Department of Geriatrics, Tampere University Hospital, P.O. Box 2000, 33521 Tampere, Finland

**Keywords:** Emergency medical services, Treatment limitations, Ethics, Nursing home, DNACPR, Decision-making, Limitation of medical treatment, Prehospital physicians, Anaesthesiology, HEMS

## Abstract

**Background:**

We investigated paramedic-initiated consultation calls and advice given via telephone by Helicopter Emergency Medical Service (HEMS) physicians focusing on limitations of medical treatment (LOMT).

**Methods:**

A prospective multicentre study was conducted on four physician-staffed HEMS bases in Finland during a 6-month period.

**Results:**

Of all 6115 (mean 8.4/base/day) paramedic-initiated consultation calls, 478 (7.8%) consultation calls involving LOMTs were included: 268 (4.4%) cases with a pre-existing LOMT, 165 (2.7%) cases where the HEMS physician issued a new LOMT and 45 (0.7%) cases where the patient already had an LOMT and the physician further issued another LOMT. The most common new limitation was a do-not-attempt cardiopulmonary resuscitation (DNACPR) order (n = 122/210, 58%) and/or ‘not eligible for intensive care’ (n = 96/210, 46%). In 49 (23%) calls involving a new LOMT, termination of an initiated resuscitation attempt was the only newly issued LOMT. The most frequent reasons for issuing an LOMT during consultations were futility of the overall situation (71%), poor baseline functional status (56%), multiple/severe comorbidities (56%) and old age (49%). In the majority of cases (65%) in which the HEMS physician issued a new LOMT for a patient without any pre-existing LOMT, the physician felt that the patient should have already had an LOMT. The patient was in a health care facility or a nursing home in half (49%) of the calls that involved issuing a new LOMT. Access to medical records was reported in 29% of the calls in which a new LOMT was issued by an HEMS physician.

**Conclusion:**

Consultation calls with HEMS physicians involving patients with LOMT decisions were common. HEMS physicians considered end-of-life questions on the phone and issued a new LOMT in 3.4% of consultations calls. These decisions mainly concerned termination of resuscitation, DNACPR, intubation and initiation of intensive care.

**Supplementary Information:**

The online version contains supplementary material available at 10.1186/s13049-022-01002-8.

## Introduction

Emergency medical service (EMS) personnel treat patients in varying circumstances with the primary aim to save lives. EMS personnel need to identify and treat seriously ill or injured patients and convey them rapidly to the hospital. Life-sustaining therapies (LST) often need to be initiated promptly in a prehospital setting to ensure a chance for meaningful recovery [1]. It is equally important to identify patients who would not benefit from aggressive treatments because of their overall health state or the characteristics of the acute situation [2]. In such cases, aggressive treatment may cause more suffering for these patients, and hence a palliative approach would be preferable.

The number of EMS missions has increased in Europe and in Australia in recent decades [3, 4] and EMS personnel encounter more aged citizens, multimorbid patients [5] and patients in nursing homes (NHs) [6]. A notable proportion (8–15%) of out-of-hospital cardiac arrests (OHCAs) requiring cardiopulmonary resuscitation (CPR) occur in NHs, and the trend is increasing [7–10]. In Finland, Helicopter Emergency Medical Services (HEMS) physicians provide assistance and treatment recommendations to paramedics both on-scene and through phone consultations in various prehospital situations. One of those situations is determining the limitation of medical treatment (LOMT). Following European Resuscitation Council Guidelines, EMS personnel can withhold LST in situations where there are secondary signs of death, obviously lethal trauma or a valid do-not-attempt-cardiopulmonary-resuscitation (DNACPR) order; other LOMTs in prehospital setting are issued by HEMS physicians [1]. Treatment practices vary geographically, and decisions on treatment and LOMT may be ethically challenging[11].

For example, across Scandinavia EMS personnel have the ability to consult with EMS physicians when they need advice [12–15], and according to a recent study HEMS physicians were consulted in 24% of all EMS missions in Finland [15]. Yet, little is known about the content of those consultation calls. The aim of this study was to investigate LOMTs during EMS paramedic-initiated consultation calls to HEMS physicians. We specifically examined the frequency and content of the LOMTs, the reasons why new LOMTs were issued, the amount and quality of the information available when making decisions on treatment and the mortality of patients with LOMTs.

## Methods

### Design and setting

This prospective, observational multicentre study on consultation calls was performed on four physician-staffed HEMS bases in Finland (Turku, Tampere, Oulu and Kuopio). The study follows the Strengthening the reporting of observational studies in epidemiology (‘STROBE’) guidelines [16].

The Finnish EMS system is three-tiered, and all EMS units are dispatched by the national emergency dispatch centre. The first tier includes the first-responding units, mainly staffed by lay rescuers equipped with automated external defibrillators. The second tier consists of both basic life support units staffed with emergency medical technicians or firefighters and advanced life support units staffed with paramedics. The third tier is physician-staffed (H)EMS units, which are dispatched to aid the most high-risk patients. There are five physician-staffed HEMS units in Finland that operate with a ground unit or a helicopter in the vicinity of university hospitals 24 h a day and reach 75% of the Finnish population in 30 min. In this study, we included four HEMS units that used a common database for medical records; the catchment areas for highly specialised medical care of the four included bases serve approximately 3.78 million inhabitants (70% of the population), and the physicians are generally experienced anaesthesiologists [6].

Most EMS missions are handled by EMS personnel, but they can call (H)EMS physicians when supervision or advice are needed. Local standard operation protocols define consultation practices. EMS personnel need to consult a physician if a medical treatment by standard protocol has been given on the scene and the patient’s condition does not improve. Typically, the (H)EMS physician is consulted on critically ill or injured patients with a vital dysfunction in situations when a physician-staffed (H)EMS unit is not dispatched to the scene, but the unit may also decide to join the mission by paramedic consultation. Medical records are electronically available for HEMS physicians only at the HEMS base; if information from medical records is needed while on-scene, the HEMS physicians can contact the on-call physicians in the hospitals. Only HEMS units use the common FinnHEMS database; other physician-staffed EMS units report their consultation calls to local medical records. This study focused on consultation calls to HEMS physicians, and did not include all EMS personnel-initiated consultation calls.

In Finland, health care facilities (HCFs) of which HEMS physicians may receive consultation calls consist mainly of municipal primary health care centres but also small hospitals. In primary health care centres there are outpatient clinics and wards with general practitioners and facilities for laboratory testing and basic X-ray imaging during office-hours. The inpatient wards serve patients in postacute care, rehabilitation and palliative care. There are various types of NHs: both public and private homes and institutions staffed with health care professionals assisting residents dependent on help in activities of daily living due to dementia, old age or multimorbidity [17]. According to the Finnish law, patients should be treated according to their will. Patients can document an advance directive in which they express their preferences regarding treatment decisions anticipating situations they are unable to communicate. Advance directive is documented in medical records and to be fulfilled, it should meet the criteria of existence, validity and applicability [2]. Indeed, if EMS personnel find a valid advance directive containing ‘DNACPR’, CPR should not be performed or continued. However, if the patient insists treatment that is not medically justified or acceptable, the physician’s judgement overrules the patient’s will. If advance directive is unclear or not known in the case of acute critical illness, the patients are offered the treatments that are medically justified. Physician assisted death is not permitted in Finland.

### Data collection

Consultation calls to HEMS physicians occurring between September 6, 2017 and March 6, 2018 were obtained from the FinnHEMS database. These calls represent 51% of all recorded events during the observation period. The remaining 5895 events represent missions where HEMS physician was on scene, and they have been analysed in another study [18]. In the present study, we focused to LOMT made by phone when the HEMS physician didn’t encounter the patient personally. The electronic database includes data on HEMS missions, consultations calls and medical records from HEMS missions [19]. For the purposes of this study, a questionnaire (study sheet) was created in the FinnHEMS database. The study sheet contained questions about the content and reasoning of the new LOMT and the quality of information available when making treatment decisions. HEMS physicians completed the study sheet when documenting a consultation call in the database. Consultation calls were defined to be associated with an LOMT if the physician (1) identified that the patient had a pre-existing LOMT, (2) issued a new LOMT or (3) identified the patient as having a pre-existing LOMT and issued a new one on the phone. In this study setting, consultation calls in which a new LOMT was pondered but not issued were excluded. Consultation calls without a (or with an incorrectly completed) study sheet were excluded from the analysis. When multiple consultation calls were observed regarding a unique patient, we included the first call in the survival analysis. The mortality rate up to November 6, 2018 was retrieved from The Finnish Population Register Centre. The study sheet is presented in Additional file 1.

### Definitions of LOMTs

In this study, ‘DNACPR’ included the decision to withhold further CPR attempts after the return of spontaneous circulation. A decision to discontinue an on-going resuscitation attempt was coded as ‘termination of resuscitation’ (ToR). ‘No intubation’ was defined as no endotracheal intubation. ‘Not eligible for intensive care’ (NEIC) meant withholding all treatments that the HEMS physician perceived as intensive care, such as invasive monitoring, endotracheal intubation, mechanical ventilation or drugs that demand intensive care unit (ICU)-level surveillance. If the patient should be transported to a municipal primary HCF for the primary care, the issued LOMT was ‘no tertiary hospital transfer’. Limiting the treatment with ‘no transfers’ meant that the patient would stay in a private home or in an NH with basic care.

### Data analysis

The main outcome variable was an identified pre-existing and/or new LOMT, and secondary outcome variables were the reasons for new LOMT, information available when making treatment decisions and survival measured as days from the consultation call. In addition, we analysed characteristics of the patients and the situations. Groups presented with frequencies and percentages were compared with a chi-square test and Fisher’s exact test when appropriate. Groups presented with medians (Q1–Q3) were compared with a Mann–Whitney *U*-test and Kruskal–Wallis test. The survival between independent and mutually exclusive groups was described with a Kaplan–Meier curve and tested with Log-Rank test. A *p*-value < 0.05 was considered statistically significant, and all tests were two-sided. IBM SPSS version 27 was used for the analyses (Armonk, NY; IBM Corp).

### Ethics

The study protocol was approved by the Ethics Committee of the Tampere University Hospital (Approval no: R15048 on March 17, 2015) and by the National Institute for Health and Welfare (THL/861/5.05.00/2015 on November 11, 2015) that also granted a permission to collect data on all consultation calls from medical records i.e. the FinnHEMS database. Due to the retrospective and register-based design of the study, the need for informed consent was waived. The 57 HEMS physicians received verbal instructions and an information letter about the study and their participation in the study was voluntary.

## Results

### Consultation calls associated with LOMT

**Fig. 1:**
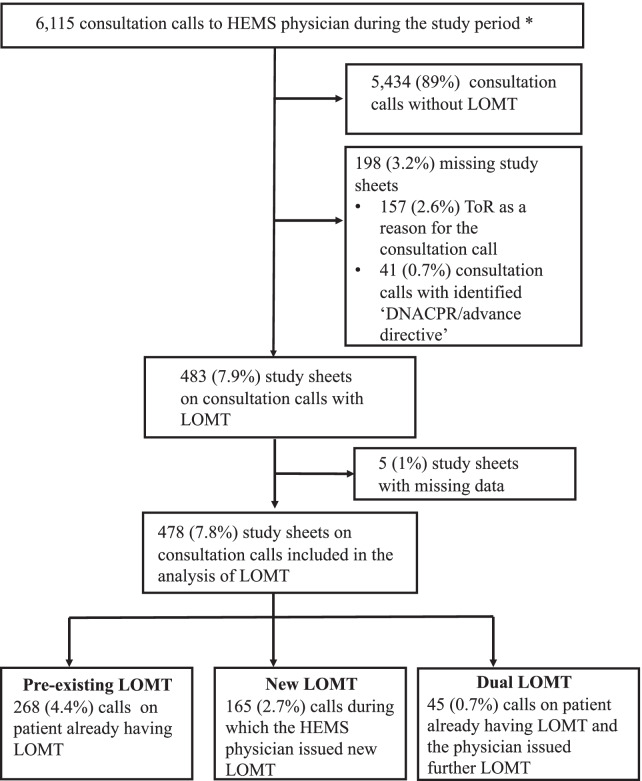
Data collection. *Patient’s unique civil registration number was known in 5330 (87%) consultation calls and these regarded 5061 unique patients

In total, there were 6115 consultation calls (approximately 8.4 calls/day/base) during the study period. There were 483 (7.9%) consultation calls associated with an LOMT, of which 478 (99%) were included in the final analysis (Fig. 1). Study sheets were filled by 52 different HEMS physicians (corresponding to 91% of all HEMS physicians at included bases). The demographic data of the consultation calls are shown in Table 1 and the content of LOMTs in Table 2. There were 313 (5.1%) consultation calls involving a pre-existing LOMT, of which 93% were ‘DNACPR’ and/or ‘NEIC’. HEMS physicians issued a new LOMT in 210 (3.4%) consultation calls, of which 45 were made for patients who already had an LOMT. In 49 (23%) calls involving a new LOMT, ToR was the only newly issued LOMT.

**Table 1:** Baseline characteristics of consultation calls to HEMS physicians

Consultation calls for EMS patients, data from medical records^a^	No LOMT		Pre-existing LOMT		New LOMT		Dual LOMT		
	N = 5632	%	N = 268	%	N = 165	%	N = 45	%	*P*
*Time of the call*									0.001
Day (8 a.m.–4 p.m.)	2214	39	116	43	87	53	21	47	
Evening (4 p.m.–12 p.m.)	2172	39	104	39	44	27	10	22	
Night (12 p.m.–8 a.m.)	1246	22	48	18	34	21	14	31	
*Reason for consultation*									< 0.001
Treatment instructions	3353	60	178	66	106	64	34	76	
Destination of further admission	802	14	43	16	8	4.8	4	8.9	
Pain medicine	481	8.5	10	3.7	0		0		
ECG interpretation	416	7.4	10	3.7	0		0		
Non-conveyance	356	3.6	12	4.5	1	0.6	1	2.2	
End of resuscitation attempt	157	2.8	12	4.5	47	29	3	6.7	
Other	67	1.2	3	1.1	3	1.8	3	6.7	
*Dispatch code*^*b*^									< 0.001
Arrythmia	1169	21	51	19	12	7.3	9	20	
Chest pain	1080	19	29	11	2	1.2	2	4.4	
Dyspnea	546	9.7	76	28	33	20	11	24	
Falling (not dropping)	306	5.4	6	2.2	3	1.8	1	2.2	
Stomach pain	274	4.9	6	2.2	2	1.2	1	2.2	
Cardiac arrest	262	4.7	11	4.1	66	40	5	11	
Convulsions	250	4.4	14	5.2	2	1.2	4	8.9	
Stroke	228	4	18	6.7	5	3	1	2.2	
Unconsciousness	184	3.3	23	8.6	27	16	9	20	
Other illness	178	3.2	21	7.8	1	0.6	0		
*Gender*									< 0.001
Male	2870	51	106	40	83	50	20	44	
Female	2497	44	155	58	67	41	25	56	
Missing data	265	4.7	7	2.6	15	9.1	0		
Age Median (Q1–Q3)	67	(48–79)	84	(75–90)	80	(70–89)	85	(78–92)	< 0.001
Children under 18 years	412	7.3	2	0.7	0		0		< 0.001
*Location of the patient*									< 0.001
Home/public/work	5078	90	95	35	96	58	11	24	
Nursing home	282	5	150	56	50	30	23	51	
Primary health care facility	188	3.3	18	6.7	13	7.9	8	18	
Hospital	66	1.2	4	1.5	6	3.6	3	6.7	
Other	18	0.3	1	0.4	0		0		
*Anamnesis*^*c*^									
Previously healthy	651	12	0		3	1.8			< 0.001
DNACPR/Advance directive	41	0.7	150	56	0		29	64	< 0.001
Hypertension	1125	20	71	27	41	25	16	36	0.002
Coronary artery disease	730	13	57	21	22	13	5	11	0.002
Diabetes	617	11	38	14	16	9.7	3	6.7	0.271
Atrial fibrillation (chronic)	501	8.9	57	21	22	13	8	18	< 0.001
Asthma/COPD	445	7.9	37	14	15	9.1	5	11	0.006
Cardiac insufficiency	302	5.4	56	21	20	12	11	24	< 0.001
Dementia	211	3.7	65	24	32	19	11	24	< 0.001
Substance abuse	175	3.1	0		6	3.6	0		0.002
TIA/stroke	155	2.8	24	9	11	6.7	7	16	< 0.001
Mental health disorder	133	2.4	4	1.5	5	3	3	6.7	0.168
Epilepsy	124	2.2	11	4.1	4	2.4	0		0.200
Another diagnosed illness	987	18	86	32	32	19	10	22	< 0.001

HEMS, Helicopter Emergency Medical Service, DNACPR, Do not attempt cardiopulmonary resuscitation, COPD, Chronic Obstructive Pulmonary Disease, TIA, Transient Ischaemic Attack

^a^ 5 consultation calls with LOMT excluded from analysis (Fig. 1)

^b^ Only the 10 most common codes out of the 50 codes observed during the study period are shown

^c^ The sums on patients with comorbidities exceed n = 6110 because many patients may have had several comorbidities

**Table 2:** The frequency and content of LOMT in consultation calls to HEMS physicians

The frequencies and contents of LOMT	N	%
***A**** Pre-existing LOMT (N* = *313)*		
The frequency of different pre-existing LOMT^a^		
DNACPR	300	96
NEIC	59	19
No tertiary hospital admission	2	0.6
No transfers	4	1.3
Other^b^	16	5.1
***B**** New LOMT (N* = *210)*		
The frequency of different new LOMT^c^		
DNACPR	122	58
NEIC	96	46
No intubation	67	32
ToR	54	26
No tertiary hospital admission	12	5.7
No transfer	12	5.7
Other^d^	24	11.4

LOMT, Limitation of medical treatment, HEMS, Helicopter Emergency Medical Service, DNACPR, Do-not-attempt-cardiopulmonary resuscitation, NEIC, Not eligible for intensive care, ToR, Termination of resuscitation

^a^In 64 consultations the patient had multiple pre-existing LOMT

^b^The category ‘other’ included three consultation calls in which the patient had an advance directive, five consultation calls on patients with palliative care decision and one call on patient with ‘allow natural death’ decision issued by a general practitioner

^c^In 108 consultation calls multiple new LOMT were issued

^d^The category ‘other’ included nine consultation calls in which the LOMT was the decision to admit the patient to the secondary hospital instead of tertiary hospital for further treatment

**Fig. 2:**
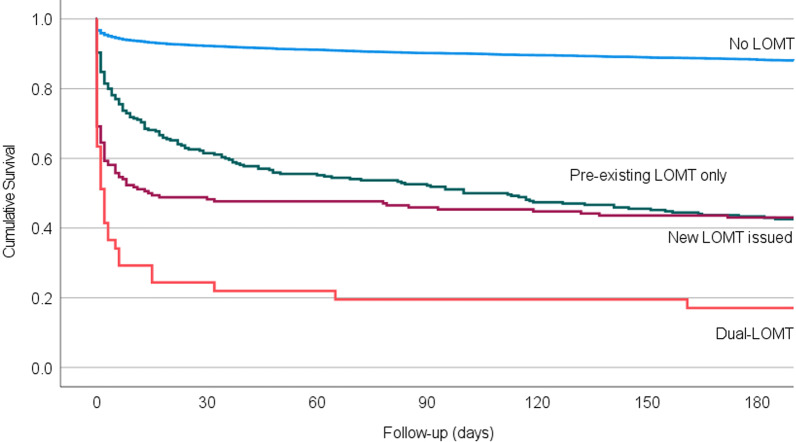
The 180-day survival of the study cohort including 5061 unique patients. Among the patients concerned in the consultation calls, 4671 (92%) had no limitation in medical treatment (LOMT), 233 (4.6%) had a pre-existing LOMT, 119 (2.4%) had a new LOMT and 38 (0.8%) belonged to an independent group of patients having pre-existing LOMT but to whom HEMS physicians issued further LOMTs (‘Dual LOMT’)

In consultation calls associated with an LOMT, the patients were older, more often in an HCF or NH and had more comorbidities, especially dementia and cardiovascular diseases (Table 1). When the HEMS physician issued a new LOMT during the consultation call, 32% of the patients died on the same day, and 66% died within a week from the consultation call (*p* < 0.001) (Fig. 2). In most consultation calls (n = 108/165, 66%) in which an HEMS physician issued a new LOMT during the call, the HEMS physician believed that the patient should have already had an LOMT. In the remaining one-third (n = 57/165, 35%) of cases, the patient had experienced trauma or an unexpected acute deterioration: The reason for consultation was most commonly ToR (n = 30/57, 53% vs. n = 17/108 16%, *p* < 0.001), and the patients were younger (median 74 vs. 84 years, *p* < 0.001) and often located in a private home or in a public location (n = 49/57, 86% vs. n = 47/108, 44%, *p* < 0.001). Of 478 consultation calls involving an LOMT, 60 (13%) were related to an HEMS mission. In almost all of these missions (n = 58), the HEMS unit was cancelled due to futility or LOMT (cardiac arrest n = 26, unconsciousness n = 22, other n = 10).

### Reasons for new LOMT

The most common single reason for a new LOMT was the futility of the overall situation (18%) (Table 3). In cases where age was selected as a reason for the LOMT, the median age of the patient was 89 (min–max 74–104) years. In cases in which poor baseline functional status was selected as a reason for the LOMT, the patient was either located in an HCF or NH (71%) or otherwise needed help in activities of daily living.

**Table 3:** The reasons that HEMS physicians issued a new LOMT during a consultation call

Reasons for new limitation of medical treatment	All n = 210	%
*The frequency of different reasons for a new LOMT*^*a*^		
Futility of the overall situation	150	71
Multiple/severe comorbidities	118	56
Poor baseline functional status	117	56
Old age	103	49
Pre-existing LOMT or advance directive	33	16
Other	14	6.7

HEMS, Helicopter emergency medical service; LOMT, Limitation of medical treatment

^a^In 159/210 (76%) consultation calls with a new LOMT, the HEMS physician selected multiple reasons for the LOMT decision

### *Information available when making decisions regarding treatment *via* phone*

In 39% of the consultation calls, the decisions were based entirely on the information given by the EMS personnel on-scene (Table 4). All consultations concerning NH patients lacked any pre-existing advance care plan with emergency care plans. Every other (n = 102/210, 49%) new LOMT was issued during a consultation call for patients in an HCF or NH. During these calls, the NH staff was unfamiliar with the resident’s comorbidities in 21% (n = 21/102) of cases, baseline functional capacity in 15% (n = 15) of cases and pre-existing LOMT in 22% (n = 22) of the cases.

**Table 4:** The information available when HEMS physicians made decisions on treatment and issued a new LOMT

Type of information available	All n = 478	%	New LOMT n = 210	%
*Information from EMS situation*	471	99	206	98
Anamnesis via EMS personnel	471	99	206	98
Measured vital parameters	399	83	160	76
ECG	151	32	45	21
*Information from a person other than the EMS personnel*^*a*^	117	24	79	38
Nurse familiar with the patient	59	12	39	19
Nurse unfamiliar with the patient	9	1.9	8	3.8
Attending physician in nursing home	6	1.3	4	1.9
Another physician	15	3.1	10	4.8
A relative/proxy	31	6.5	22	10
*Information on any pre-existing LOMTs*	291	61	35	17
*Medical records*	187	39	60	29
Tertiary hospital medical records	182	38	57	27
Primary care hospital/health care facility medical records	3	0.6	0	0
Nursing home client’s medical records	7	1.5	3	1.4
Emergency care plan	0	0	0	0
Kanta-service^b^/National electronic medical records	1	0.2	1	0.5
Medication list	124	26	45	21
Medication list without medical records	29	6.1	17	8.1
Information available only from paramedics on scene^c^	85	18	82	39

HEMS, Helicopter Emergency Medical Service; LOMT, Limitation of medical treatment

^a^Not EMS personnel; for example, a relative/proxy, nursing home staff, physician in tertiary hospital

^b^National archive of health and social welfare information contains up to date records from both the private and public sectors. The users of the Kanta services include citizens, healthcare services, social welfare services and pharmacies

^c^Information on medical records, medication lists, pre-existing LOMTs or from any person other than EMS personnel on scene was not available

HEMS physicians mostly made decisions about new LOMTs without seeking a second opinion (n = 186/210, 89%). In 20 (10%) cases, the HEMS physicians discussed the decision via phone with another physician from a tertiary hospital or with another HEMS physician. Discussions with HCF/NH physicians were rare (n = 3, 1%). When making treatment decisions for patients with a pre-existing LOMT, the HEMS physicians received information about the previously issued LOMT in 93% (n = 291/313) of cases. In the remaining 7% of cases, the HEMS physician reported having received this information after decisions were already made, usually by reading it from the medical records when documenting the consultation call.

## Discussion

This prospective, observational multicentre study investigated 6115 EMS personnel-initiated consultation calls to HEMS physicians, with a special interest in LOMTs. Answering consultation calls is an important part of HEMS physicians’ daily tasks, and 8% of these calls were associated with an LOMT. The incidence of LOMTs is increasing in Europe [20, 21]. In a French study, advance directives were available for 7.5% of OHCA patients [13], and in a recent study from USA, 9.9% of EMS-attended OHCA patients had a DNACPR order [10]. In our study, patients’ pre-existing LOMTs were mainly ‘DNACPR only’ (75%), and no advance care plans with emergency care plans were reported [2, 22]. New LOMTs were issued in 3.4% of consultation calls, and half of them were made for patients in an HCF or NH. The most frequent new LOMT was a ‘ToR’ (23%), but ‘DNACPR’, ‘NEIC’ and ‘no intubation’ were also common.

We found that HEMS physicians issued LOMTs in extremely futile situations. According to ERC guidelines [1], withdrawal from CPR should be considered when there is no return of spontaneous circulation, no shocks are administered and EMS personnel did not witness the arrest. Resuscitation attempts can be terminated if there has been asystole continuously despite 20 min of advanced life support with an absence of a reversible cause of cardiac arrest [1]. The large proportion of ToR decisions explains the poor survival in the study population: one-third of patients with a new LOMT died on the same day of the consultation call. Notably, in two-thirds of the consultation calls involving new LOMTs the HEMS physician felt that the patient’s fragile condition should have ethically mandated that the treatment limitation be issued earlier. One possible explanation for this is that the LOMT did exist, but information on these advance care plans was not available on-scene during the call, which has been reported in other studies [9, 13]. It is possible that the patient records were not available or that the family or nursing staff were unable to share this information. The criteria to initiate LST in prehospital settings are the same as admission criteria for intensive care in hospitals, but the diagnostic possibilities on-scene and access to medical records are limited. We found only one study analysing the reasons for ICU refusal rather than ICU admission [23]. The reasons to limit LST and refuse ICU admission were higher age, underlying disease, NH residency, pre-existing cognitive impairment, admission for medical reasons, sepsis, acute cardiac failure or acute central neurologic illness. In that study, 59% of the decisions to forego LST for ICU-refused patients were made via phone[23].

During the study period, we observed 5895 HEMS missions and 6115 consultation calls, although parts of the calls were related to the missions [18]. Compared to an earlier Scandinavian study, only 23% of HEMS units’ events were phone calls [12]. In Finland, HEMS physicians were consulted in 39% of non-conveyance situations [15]. An interesting feature of our study was that the HEMS physicians seldom discussed LOMT decisions with another physician, which needs further consideration. This is probably due to Finnish HEMS physicians’ extensive work experience in anaesthesiology and intensive care and the high proportion of ToR decisions. This is very different from a French study [24], which reported that the issuance of a new LOMT in the field was common, but the physicians consulted another physician in 59% of the cases. In this study, 14% of calls concerned patients in an HCF or NH, and half of the new LOMTs were issued for those patients, often during daytime. There should be an attending physician who has access to the patient’s medical records and/or customer information and is reachable by phone at least during office hours in HCFs and NHs. When an HEMS physician issues a new LOMT during a consultation call, the information should be transmitted to the attending physician to plan the follow-up care and make an advance care plan.

There is a general consensus regarding the need for emergency care plans over limitations in treatment only [25]. However, it has been found that the EMS system helps to overcome deficiencies in end-of-life care: The report of the ﻿National Supervisory Authority for Welfare and Health states that EMS units are increasingly dispatched to treat patients in NHs due to inadequate advance care plans [26]. In addition, the Ministry of Social Affairs and Health’s report on the status of the palliative care in Finland acknowledges that EMS often responds to the sudden care needs of patients in end-of-life care [27]. EMS is the only nationwide societal health care system that provides all levels of health care 24/7. Waldrop et al. identified care-related, psychological and organisational reasons that prehospital providers are called to NHs at the end of life [28]. If advanced care planning is concentrated mainly on treatment restrictions without an emergency care plan [22], when the patient suddenly deteriorates, EMS participation is usually needed to take the responsibility for clinical decision-making about when to proceed to palliative care. The patient’s family members or NH staff usually need diagnostic measures and support [10, 13]. Sometimes, prehospital providers do not understand the pre-existing LOMT or need treatment instructions on how to treat critically ill patients with LOMTs [29]. The dying process should not be medically lengthened; instead, in situations where the end of life is approaching the goals of care should be palliation and dignity.

End-of-life care decisions are often difficult to make before the acute severe illness or injury. Societal service systems have difficulties handling these acute problems during on-call hours due to challenges in information transmission, work culture and work organisation. Thus, the current system places responsibility for acute decision-making upon HEMS physicians [30]. In situations where the new LOMT is issued on the phone by an HEMS physician and the patient does not die immediately, it may be challenging to ensure good continuity of care without contact with the physician responsible for the follow-up care. In view of the increased workload of the EMS system, decision makers in health care systems should actively consider the organisation and accessibility of end-of-life care services in acute situations.

## Strengths and limitations of the study

To the best of our knowledge, this is the first study on pre-hospital consultation calls concerning LOMTs. The strengths of this study are the prospective multicentre design and feasible data collection; the study sheet was available in the same electronic database that was used for documentation of the consultation calls. The material is comprehensive, and the study has a nationwide coverage. Patient’s unique civil registration number was missing in 13% of consultation calls and in addition three patients in the ‘no LOMT’ group were lost from follow-up since registered abroad. However, the practices of HEMS physicians might vary in terms of why or how actively they issue LOMTs [31–33]. This study did not record cases where a new LOMT was considered but not issued. In addition, it is possible that some HEMS physicians do not perceive the termination of a futile resuscitation attempt as an LOMT, and in such a situation they may have been unlikely to complete the study sheet. Thus, the true incidence of end-of-life questions in prehospital settings may be even higher. The results of this study cannot be generalised to other countries because the EMS and health care systems, end-of-life arrangements and NHs may be remarkably different from the Finnish systems.

## Conclusion

Consultation calls to HEMS physicians concerning LOMTs are common. HEMS physicians advise EMS personnel on the phone regarding end-of-life questions as well, and in 3.4% of consultation calls they issue a new LOMT. These decisions mainly concern ToR, DNACPR, intubation and initiation of intensive care. Further research is recommended on the continuity of care of patients with a prehospital LOMT: for example a prospective study on how the information of LOMT and/or admission to palliative care should be communicated to the NH physician or a follow-up study on what happens to patients with LOMT in hospital after they are admitted to emergency department.

## Supplementary Information

**Additional file 1**. The study sheet with English translations.

## Data Availability

Please contact the author for data requests.

## Abbreviations

CPR: Cardiopulmonary resuscitation
DNACPR: Do not attempt cardiopulmonary resuscitation
EMS: Emergency medical services
HCF: Health care facility
HEMS: Helicopter emergency medical services
ICU: Intensive care unit
LOMT: Limitation of medical treatment
LST: Life-sustaining therapies
NEIC: Not eligible for intensive care, NH: nursing home, OHCA: out-of-hospital cardiac arrest, ToR: termination of resuscitation
