# Supplementary material for: Advising and limiting medical treatment during phone consultation: a prospective multicentre study in HEMS settings
Source: Scand J Trauma Resusc Emerg Med. 2022 Mar 9;30:16. doi: 10.1186/s13049-022-01002-8 (PMC8905861; doi:10.1186/s13049-022-01002-8)
Supplement: Supplementary file 1 — Additional file 1. The study sheet with English translations. [file 13049_2022_1002_MOESM1_ESM.pdf]

Hoidonrajaustutkimus [R4-486]

Tehtävän / konsultaation tiedot

Tyyppi

Päivämäärä

Hälytyskoodi

Nimi

Henkilötunnus

Hoidonrajaus

Tutkimuskysymykset

1) Oliko potilaalla ennestään hoidonrajauksia (valitse kaikki olemassa olevat hoidonrajaukset)?

- [ ] Ei
- [ ] Ei elvytetä
- [ ] Ei tehohoitoa
- [ ] Ei sairaalahoitoa (esim. hoidetaan terveyskeskustasolla)
- [ ] Sairastuessa ei siirtoa muualle (hoidetaan hoitolaitoksessa/kotona)
- [ ] Muu, mikä?

2) Sait konsultaatiopuhelun potilaasta, jota et tavannut henkilökohtaisesti. Mitä tietoja sinulla oli käytettävissäsi hoitopäätöksiä tehtäessä? i

Potilaskertomus: Ei Kyllä

- [ ] Perusterveydenhuollosta
- [ ] Hoivakodista tms.
- [ ] Erikoissairaanhoidosta
- [ ] KANTA-arkistosta
- [ ] Suunnitelma toiminnasta akuutissa tilanteessa
- [ ] Muu, mikä?

Lääkelista: Ei Kyllä

Tiedot ensihoitotilanteesta: Ei Kyllä

- [ ] Oma kliininen tutkimus
- [ ] Ensihoitajan antamat esitiedot
- [ ] Vitaaliarvot
- [ ] EKG

Tieto aiemmista, olemassa olevista hoidonrajauksista omaa nykyistä hoitopäätöstä tehdessäsi: Ei Kyllä i

- [ ] Ei elvytetä
- [ ] Muu hoidonrajaus

Esitietoja muulta kuin ensihoitoon kuuluvalta henkilöltä: Ei Kyllä

- [ ] Hoitajalta, joka tunsi potilaan ennestään
- [ ] Hoitajalta, joka ei tuntenut potilasta ennestään
- [ ] Hoitavalta lääkäriltä
- [ ] Muulta lääkäriltä i
- [ ] Omaiselta
- [ ] Muu, kuka? i

Muita tietoja, mikä tai kuka?

3) Millä tavalla rajasit hoitoa (valitse kaikki rajaukset jotka teit)?

- [ ] Ei elvytetä i
- [ ] Lopetetaan elvytys i
- [ ] Ei tehohoitoa i
- [ ] Ei sairaalahoitoa i
- [ ] Ei siirretä tilanteesta huolimatta muualle i
- [ ] Ei intuboida
- [ ] Muu, mikä?

4) Minkälaisia potilaaseen tai tilanteeseen liittyviä tekijöitä oli tekemäsi hoidonrajauksen taustalla? Valitse kaikki tekijät, joilla perustelit tehdyn hoidonrajauksen.

- [ ] Perussairaudet
- [ ] Alentunut toimintakyky
- [ ] Korkea ikä
- [ ] Tapaamishetkellä huonoennusteiselta vaikuttava kokonaistilanne i
- [ ] Aiemmat jo olemassa olleet hoidonrajaukset
- [ ] Muu, mikä?

5) Tämä potilas kohdattiin hoitolaitoksessa. Oliko hän:

- ( ) Pitkäaikaishoidossa samassa hoitolaitoksessa, jonne ensihoito hälytettiin
- ( ) Pitkäaikaishoidossa muussa hoitolaitoksessa, kuin mihin ensihoito hälytettiin
- ( ) Lyhytaikaisessa tai tilapäisessä hoidossa hoitolaitoksessa, jonne ensihoito hälytettiin
- ( ) Muu kuin hoitolaitoksen potilas (työntekijä tai omainen tms.)
- ( ) En tiedä

6) Osasiko hoitolaitoksen henkilökunta mielestäsi kertoa tavattaessa/puhelimessa potilaan:

Perussairauksista:	Ei	Kyllä	En tiedä
Toimintakyvystä:	Ei	Kyllä	En tiedä
Olemassa olevista hoidonrajauksista:	Ei	Kyllä	En tiedä

7) Olitko henkilökohtaisesti yhteydessä omaisiin informoidaksesi heitä hoidonrajauspäätöksestä?

En | Kyllä

8) Keskustelitko hoidonrajauksen tekemisestä toisen lääkärin kanssa?

En | Kyllä

- [ ] Toinen ensihoitolääkäri
- [ ] Hoitolaitoksen lääkäri
- [ ] Erikoissairaanhoidon lääkäri
- [ ] Muu, mikä?

9) Olisiko potilaalla mielestäsi pitänyt olla hoidonrajauksia olemassa ennen nykyistä hälytystehtävää?

( ) Ei ( ) Kyllä

Kommentit

Muuta mieleen tulevaa...

Tallenna tiedot | Poista keskeneräinen

4) Minkälaisia potilaaseen tai tilanteeseen liittyviä tekijöitä oli tekemäsi hoidonrajauksen taustalla? Valitse kaikki tekijät, joilla perustelit tehdyn hoidonrajauksen.

- [ ] Perussairaudet
- [ ] Alentunut toimintakyky
- [ ] Korkea ikä
- [ ] Tapaamishetkellä huonoennusteiselta vaikuttava kokonaistilanne i
- [ ] Aiemmat jo olemassa olleet hoidonrajaukset
- [ ] Muu, mikä?

5) Tämä potilas kohdattiin hoitolaitoksessa. Oliko hän:

- ○ Pitkäaikaishoidossa samassa hoitolaitoksessa, jonne ensihoito hälytettiin
- ○ Pitkäaikaishoidossa muussa hoitolaitoksessa, kuin mihin ensihoito hälytettiin
- ○ Lyhytaikaisessa tai tilapäisessä hoidossa hoitolaitoksessa, jonne ensihoito hälytettiin
- ○ Muu kuin hoitolaitoksen potilas (työntekijä tai omainen tms.)
- ○ En tiedä

6) Osasiko hoitolaitoksen henkilökunta mielestäsi kertoa tavattaessa/puhelimessa potilaan:

Perussairauksista:	Ei	Kyllä	En tiedä
Toimintakyvystä:	Ei	Kyllä	En tiedä
Olemassa olevista hoidonrajauksista:	Ei	Kyllä	En tiedä

7) Olitko henkilökohtaisesti yhteydessä omaisiin informoidaksesi heitä hoidonrajauspäätöksestä?

En | Kyllä

8) Keskustelitko hoidonrajauksen tekemisestä toisen lääkärin kanssa?

En | Kyllä

- [ ] Toinen ensihoitolääkäri
- [ ] Hoitolaitoksen lääkäri
- [ ] Erikoissairaanhoidon lääkäri
- [ ] Muu, mikä?

9) Olisiko potilaalla mielestäsi pitänyt olla hoidonrajauksia olemassa ennen nykyistä hälytystehtävää?

○ Ei ○ Kyllä

Kommentit

Muuta mieleen tulevaa...

Lomake valmis

Valmis: Ei | Kyllä

Tallenna tiedot

Poista keskeneräinen

Kangasniemi et al. Advising and limiting medical treatment during phone consultation: a prospective multicentre study in HEMS settings

The English translations to study-sheet R4-486

The study sheet was integrated in the FinnHEMS database.

To the main page of HEMS database where new HEMS missions and consultation calls to HEMS physicians were routinely documented, we had added an extra question "was this consultation call/HEMS mission associated with limitations of medical treatment?". If the physician ticked 'yes', a study sheet was then generated within the same database.

Another important question on the main page was question 'what was the location of the patient?'
_home/public/work
_hospital
_primary health care center
_nursing home
_other

The study sheet was interactive; answering 'yes' to certain question opened a new submenu on the study sheet. In addition, depending on how the physician had previously answered, some question were inactive. For example, questions concerning nursing homes and health care facilities were active only if the patient was in those.

Information of the HEMS mission/consultation call

Type (mission/consultation call) **Name**

Date **Patient's civil registration number**

Dispatch code **Limitations of medical treatment (LOMT)**

_ Pre-existing LOMT
_ New LOMT
_ Dual LOMT (both pre-existing and new LOMT)

STUDY QUESTIONS

1) Did the patient have any pre-existing LOMT (please choose all pre-existing LOMT)?
_ No
_ DNACPR
_ Not eligible for intensive care
_ No tertiary hospital admission (admission to primary health care facility if necessary)
_ No transfers if deteriorated (stays at home/ in nursing home/in primary health care facility inpatient ward)
_ Other, what? _________________

2) You received a consultation call on patient and you did not encounter the patient in personal. What kind of information you had in use while making decisions on treatment?
_ Medical records
  _ Primary health care facility medical records
  _ Nursing home client's medical records
  _ Tertiary hospital medical records
  _ Kanta-service/National electronic medical records

_Emergency care plan for acute deterioration
_ Other, what? _________________

_Medication list

_ Information from EMS situation
_Clinical examination performed by the HEMS physician (not active for consultation calls)
_ Anamnesis from a paramedic
_ Measured vital parameters
_ECG

_ Information of the existence of the pre-existing LOMT while making your own decisions on treatmetn?
_DNACPR
_Other LOMT

_ Information from a person other than the EMS provider
_ Nurse familiar with the patient
_ Nurse unfamiliar with the patient
_ Attending physician in nursing home/in health care facility
_Another physician
_ A relative/proxy
_ From another person, from whom?

_Another information, what? _____________

3) What kind of LOMT you made? Please choose all new LOMT you made.

_DNACPR
_Termination of resuscitation
_Not eligible for intensive care
_ No tertiary hospital admission (admission to primary health care facility if necessary)
_ No transfers though deterioration (stays at home/ in nursing home/in primary health care facility inpatient ward)
_ No intubation
_ Other, what? _________________

4) Which patient and/or situation related factors contributed to your decision to issue new LOMT? Please select all reasons that accounted for the LOMT.
_ Multiple/severe comorbidities
_ Poor baseline functional status
_ Old age
_ Futility of the overall situation
_ Pre-existing LOMT or advance directive
_ Other, what? _________________

Questions 5 and 6 active only for if the HEMS physician had ticked 'the patient was in nursing home/health care facility'.

5) This patient was encountered in a nursing home or in a health care facility?

_In long-term care in the same unit where the EMS was dispatched
_ In long-term care in another nursing home/health care facility than the unit were the patient was when EMS was dispatched
_In short-term care in the nursing home/health care facility to where EMS was dispatched
_ The patient was other than nursing home client/patient in health care facility (for example a employee or proxy)
_ I don't know

6) In your opinion, was the nursing home/health care facility staff able to answer questions on resident's…

**comorbidities?**	*yes/no/I don't know*
**baseline functional capacity?**	*yes/no/I don't know*
**pre-existing LOMT?**	*yes/no/I don't know*

7) Did you personally contact relatives to inform them of the LOMT decision?

yes/no

8) Did you discuss with another physician while issuing a new LOMT?

yes/no

_Another HEMS physician
_Physician in nursing home/health care facility
_Physician in tertiary hospital
_ Other, who? _________

Question nine was active for only situations when the physician had previously ticked 'new limitations' and inactive if the physician had ticked options 'Pre-existing LOMT' or 'Dual LOMT' in the beginning of the study sheet.

9) In your opinion, should the patient have had LOMT before this EMS dispatch?
yes/no

Comments
(space for free comments)

Study sheet ready
yes/no

SAVE **ERASE**
